# Nutritional status of under-five aged children of ready-made garment workers in Bangladesh: A cross-sectional study

**DOI:** 10.1371/journal.pone.0284325

**Published:** 2023-04-13

**Authors:** Sadika Haque, Dewan Abdullah Al Rafi, Nafisa Zaman, Md. Salman, Md. Abdullah Al Noman, Md. Nazmul Hoque, Lalita Bhattacharjee, Samantha Farquhar, Sabina Yasmin, Md. Mehedi Hasan, Fatema Tuj Zohora Hira, Aunjuman Ara Prithi, Shamim Ara Shammi, Bilkish Banu, Akbar Hossain

**Affiliations:** 1 Department of Agricultural Economics, Bangladesh Agricultural University, Mymensingh, Bangladesh; 2 Department of Agricultural and Resource Economics, The University of Arizona, Tucson, Arizona, United States of America; 3 Student Affairs Division, Bangladesh Agricultural University, Mymensingh, Bangladesh; 4 Senior Nutrition Advisor, Meeting the Undernutrition Challenge Programme, Food and Agriculture Organization of the United Nations, Rome, Italy; 5 Integrated Coastal Sciences, East Carolina University, Greenville, North Carolina, United States of America; 6 Socio Economics Research Division, Bangladesh Livestock Research Institute, Savar Union, Bangladesh; 7 Faculty of Social Science and Humanities, Department of Economics, Hajee Mohammad Danesh Science & Technology University, Dinajpur, Bangladesh; 8 Division of Soil Science, Bangladesh Wheat and Maize Research Institute, Dinajpur, Bangladesh; Quaid-i-Azam University Islamabad: Quaid-i-Azam University, PAKISTAN

## Abstract

**Background:**

The ready-made garment (RMG) sector is a significant contributor to the economic growth of Bangladesh, accounting for 10% of the country’s GDP and more than 80% of its foreign exchange earnings. The workforce in this sector is predominantly made up of women, with 2.5 million women working in the industry. However, these women face numerous challenges in carrying out their culturally-expected household responsibilities, including childcare, due to severe resource constraints. As a result, the children of these working women have a higher incidence of malnutrition, particularly stunted growth. This study aims to identify the factors that contribute to stunting in children under the age of five whose mothers work in the RMG sector in Bangladesh.

**Methods:**

The study collected data from 267 female RMG workers in the Gazipur district of Bangladesh using a simple random sampling technique. Chi-square tests were used to determine the associations between the factors influencing child stunting, and Multinomial Logit Models were used to estimate the prevalence of these factors.

**Results:**

The study found that the prevalence of moderate and severe stunting among the children of RMG workers living in the Gazipur RMG hub was 19% and 20%, respectively. The study identified several significant predictors of child stunting, including the mother’s education level, nutritional knowledge, control over resources, receipt of antenatal care, household size, sanitation facilities, and childbirth weight. The study found that improving the mother’s education level, increasing household size, and receiving antenatal care during pregnancy were important factors in reducing the likelihood of child stunting. For example, if a mother’s education level increased from no education to primary or secondary level, the child would be 0.211 (0.071–0.627) and 0.384 (0.138–1.065) times more likely to have a normal weight and height, respectively, than to be moderately stunted.

**Conclusion:**

The study highlights the challenges faced by working women in the RMG sector, who often receive minimal wages and have limited access to antenatal care services. To address these challenges, the study recommends policies that support antenatal care for working-class mothers, provide daycare facilities for their children, and implement a comprehensive social safety net program that targets child nutrition. Improving the socioeconomic status of mothers is also critical to reducing child malnutrition in this population.

## Background

In the last 40 years, the ready-made garment (RMG) sector has been contributing as one of the most important driving forces behind Bangladesh’s economic development, for which the country has turned out as the second-largest exporter of garment products globally [1]. The recent nature of development and urbanization has forced women to join the workforce in the formal sector but retaining with their domestic (cleaning, cooking, washing, taking care of children and elderly etc.), biological (child birth, breast feeding etc.), and cultural (leading the family while men are absent) responsibilities [2], turning their livelihood time burden more and more challenging. It is estimated that about 4.22 million workers are employed in this sector, among them 2.50 million are female [3]. Most of the RMG workers migrated from rural to urban areas, struggling with poverty in their places of origin and could not afford to continue studying [4], for which they opted to join the RMG factory as their last resort. Although the RMG industry has provided these women with the first opportunity to enter into the formal workforce, offering a substantial source of income, and economic independence, they mostly live in places of urban areas with bare minimum civic facilities (hospitals, schools, sports fields, fire stations, public transport, daycare centres etc.), have to work for more than 8 hours per day, and to make matters worse, are poorly paid (monthly pay ranges between USD $100–160) compared to their cost of basic needs [5]. Consequently, these young, inexperienced women have to compromise their health needs and be entitled to marginal health status. Many of them, at the same time, are mothers and wives, who are obligated to perform culturally-expected responsibilities in their households. Needless to mention that playing the role of both professional worker and full-time caretaker is of immense difficulty for these RMG women workers and often the health and nutrition of their children are negatively affected. Previous research has shown that child health is linked to poor access to health services, poor nutritional quality, lack of decent living conditions, shortage of adequate maternity protection, inadequate breastfeeding support, poor access to quality childcare, long working hours, and low education [6]. Yet, the nutritional status of RMG working mothers and their children has rarely been explored by researchers and policymakers.

A standard indicator of malnutrition in the case of children under the age of five is stunting which reflects the cumulative effects of poor maternal health and nutrition (like low maternal weight), frequent illness (diarrhoea, fever etc.), inappropriate feeding, food insecurity and improper care in the first 1000 days of life [7]. In addition, malnutrition hits its highest point at the age of 18 to 35 months of the child age, the complementary breast-feeding period [8]. The major consequences of stunting include poor cognitive development and educational performance, lost productivity accompanied by obesity in later childhood, and an increased risk of nutrition-related chronic diseases in adult life [9]. The rates of stunting for children under five years recorded in Bangladesh were 31% [10] and 28% [9,11,12] whereas the worldwide rate is 21.3% [13].

Different variables have been investigated as influential predictors for the stunting of <5 years of children. Zongrone et al. [14] highlighted the importance of infant and young child feeding (IYCF) practices and dietary diversity as determinants of child growth outcomes. Bairagi and Chowdhury [15] found that socioeconomic status and anthropometric indices are related to child mortality and health. Mothers’ nutritional status, and body mass index (BMI), have also been used as a predictor of child nutritional status, where some studies specifically found that the children of mothers, who have a higher BMI, are less likely to be stunted [8,16–26]. Other studies found that mothers’ education has a significant impact on a child’s nutritional status [8,16–30]. However, some authors have opposed this statement since they found mothers’ education as insignificant possibly due to the quality of education received [30–32]. Some studies indicated that a mother’s practical knowledge about nutrition might be more important than formal maternal education for child nutrition outcomes [21,33]. Some of the studies have emphasized that during antenatal care (ANC) visits, doctors and health professionals provide important facts related to child health and some clinical services, and thus better ANC facilitate improved child nutrition [26,34,35].

Households’ socioeconomic status is another predictor of stunting and nutritional status. Different authors found that a child from a higher wealth-indexed family is less likely to be stunted or malnourished because of a higher ability to buy diverse diets and health-seeking behaviour [8,19,21,35–43]. For example, a family with substantial financial and social capital can ensure an improved sanitation facility and safe drinking water, resulting in reduced stunting [8,15,27,36,37,44–46]. Similarly, improved sanitation mitigates the risk of diarrhoea which reduces the risk of malnutrition [20,44,47–53]. Previous studies also found that a large family size increases the risk of under-five child stunting because it increases food insecurity for family members [21,32,54–58]. Yet, some findings [59,60] contradict the fact that a large family size often reduces the risk of stunting because, in a large family, more people may be available to take care of the children when the mother is outside of the home for a long period.

Given the food and nutritional vulnerabilities of RMG workers and their children, along with the continued growth of the RMG sector, there is a need to understand the status of malnutrition and to scrutinize the factors affecting the nutritional status of RMG workers’ children. Therefore, this study is an attempt to recognize the social, institutional and financial factors influencing the stunted growth of under-five aged children and to provide policy recommendations.

## Methods

### Sample size and sampling technique

The population size of RMG workers is 120242 [3]. This study took the 20% portion of the population to estimate the sample size. The following method is used when the population is finite [61].


$$n=N\times \frac{\frac{Z^{2}\times p\times (1-p)}{e^{2}}}{N-1\times \frac{Z^{2}\times p\times (1-p)}{e^{2}}}$$

N = Population size (120242); Z = precession level at 95% confidence interval (1.96); p = proportion of the population (20%); e = error margin (5%).

**Fig 1:**
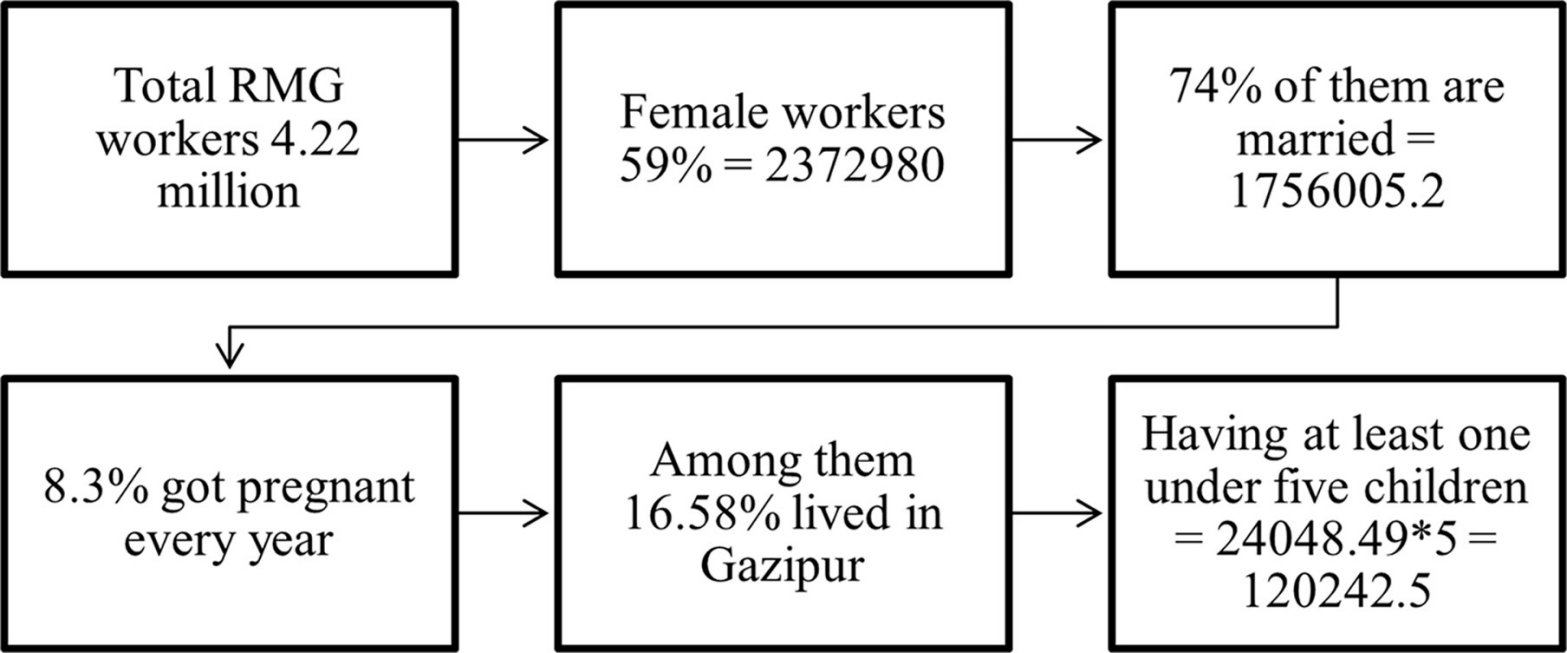
Determination of population size. Source: [3].

Through this calculation, it was found that at least 245 samples are needed to represent the population based on the above information. Based on demographic background, child health, and nutrition, a questionnaire was used to collect data from targeted respondents through a simple random sampling technique. Overall, 267 samples were collected from RMG workers who have children under five years of age. The process of calculating the population size is as follows (Fig 1):

### Data collection

Primary data was obtained from the direct field surveys, conducted in the Gazipur district between June and September 2020. These surveys focused solely on women in the RMG sector. This district was chosen as almost 60% of the RMG factories are located in Dhaka and Gazipur districts in Bangladesh [62]. A survey was conducted using a simple random sampling technique to collect data from 267 women RMG workers who have at least one biological child aged between 6 to 59 months. Anthropometry measurements of women and children were conducted using instruments of exact precision, such as digital weight measuring scales, wooden height measuring scales, and standard tape for measuring Mid-Upper Arm Circumference (MUAC). The weight of mothers and children was recorded in kilograms, and height and MUAC were recorded on a centimetres scale. The digital weight measuring scales were calibrated before each measurement, and the weight was recorded three times. The average of the three measurements was used during the analysis. Enumerators received 5-day training to provide guidelines for quality data collection and anthropometric measurements. Before the interview, respondents provided consent for themselves as well as their under-5 aged children (as their guardians of the minors) included in the study. A pretested structured questionnaire was used to collect the required data from the RMG working mothers.

### Inclusion and exclusion criteria

Data was collected on socio-demographic information that included age, wealth status, household size, household sanitation practices, education level, nutrition knowledge, health care seeking during pregnancy, childcare practices, mother and child dietary patterns, and more. The study maintained certain criteria for selecting samples while collecting data. For example, we only considered and interviewed RMG workers who accompanied their youngest, biological, and single-tone outcome child, ranging in age between 6–59 months. We avoided those who did not know the birth weight of their child, or did not have any record of birth weight, as well as those who did not allow us to measure their height, weight, and MUAC, as well as their child’s. We also did not include any pregnant women in the study.

### Variable description

#### Outcome variable

Anthropometry data (height/length and weight) and age were used to estimate the Height-for-Age (HAZ) index. WHO Multicenter Growth Reference Study (MGRS) 2006 Child Growth Standards were followed [63] and WHO Anthro version 3.2.2 was used to calculate the Z-score for HAZ. Less than -2 Standard deviation (SD) Z-score of HAZ was considered as stunted [64]. Moderate and severe stunting were defined when Z-scores for HAZ ranged between -2SD to -3SD and < −3SD respectively [65].

#### Predictor variables

Various demographic and socioeconomic characteristics of children and mothers, antenatal care status of mothers, and evidence about caregiver and daycare centre, were used as predictor variables. Variables were classified into several groups by following the standard guidelines and previous literature.

Mothers’ education was recorded based on completed years of formal schooling and further classified into different categories (no education, primary [1–5 years], secondary [6–10 years], higher secondary and more [>10 years]). To assess the mother’s nutrition knowledge, six relevant questions with binary answers were asked and assigned values 1 for “yes” and 0 for “no”. All the scores were summed up and then categorized into three groups: poor, fair, and good. The poor category was defined as the 0% to 50% range, the fair category between the 51 to 75% range, and the good category as the 76 to 100% range [66]. Women’s access to and control over financial resources describes whether the respondent mother can decide to spend her own income and/or she can spend the earning of her husband. This variable was termed as control over financial resources in McKenna et al. [67].

Antenatal care was a binary variable representing whether the mothers had received the ANC at least 4 times or not during pregnancy. Household size was measured by the number of household members currently living in the family "Guideline for Sanitation and Health" developed by WHO [68] was used to measure sanitation, which was then categorized into two classes: proper and improper. Improved sanitation was defined as the use of facilities such as piped sewer systems, septic tanks, off-set pit latrines with slab, and dual pit latrines, while unimproved sanitation included direct pit latrines with slab, direct pit latrines without slab, hanging toilets, and others. This variable is dichotomous. Birth weight was classified as a binary variable by using a threshold of 2.5 kg at the time of the baby’s birth. If a baby’s birth weight was less than 2.5 kilograms, it is then classified as low birth weight (LBW) [69]. One additional dummy variable was created to represent the caregivers’ knowledge of dietary diversity, which refers to their understanding of proper nutrition while caring for the child in the absence of the mother. A binary variable was also created to indicate whether the child was being cared for at a daycare center or home.

### Statistical analyses

Descriptive and inferential analyses were performed using STATA 17.0 software to evaluate the predictors of stunting. Descriptive analysis was used to show the frequency distribution of different variables. The Pearson Chi-square test was conducted to test the association between background characteristics and stunting prevalence. This test measures the relationship between the categorical outcome variable and the other categorical predictors.

For the inferential analysis, a logit model, specifically a multinomial logit regression, was used because the outcome variables had more than two categories [70]. The multinomial logistic model calculates the probability of each outcome as follows:

$$p_{ij}\frac{e^{\sum_{j=1}^{k}\alpha +\beta_{kj}+\varkappa_{kji}}}{\sum_{j=1}^{J}e^{\sum_{j=1}^{k}\alpha +\beta_{kj}+\varkappa_{kji}}}$$

where i represents the cases, j represents the categories, and k represents the predictor variables. The exponential of the beta coefficient, also known as the relative risk ratio (RRR), was used to explain the result of the regression [71]. Four separate models were used, with mothers’ education being categorical (based on different levels of education stages in Bangladesh) and continuous (years of schooling). Two separate models were estimated, keeping them separate. A similar technique was used for mothers’ nutrition knowledge, which was assessed based on knowledge scores and categorized as poor, fair, and good. The best-fitted model was identified based on the lowest Akaike Information Criteria (AIC) [72].

Post-estimation tests were also conducted to check the assumption of independence of irrelevant alternatives (see Supplementary Materials).

### Ethical considerations

The study protocol was reviewed and approved by the institutional review board of the Bangladesh Agricultural University Research System (BAURES) and accepted by the Technical Assistant Team (TAT) of the MUCH project of the Food and Agricultural Organization of the United Nations prior to commencing the study. Respondents were informed that the information gathered would only be used for research purposes. After providing a detailed description of the research, participants signed a written consent form, which was securely kept. To protect participants’ rights, research enumerators were trained on the research protocol and ethical principles of human subjects research. The confidentiality of information was strictly maintained, and all participants were assigned unique identifying numbers. The enumerators and the entire research team stored data in locked filing cabinets and on password-protected computers.

## Results

### Descriptive and Bivariate analysis

When the Z-score of stunting was categorized, it was found that 38% of children were stunted, with 19% being severely stunted and 19% being moderately stunted. The remaining children were either normal or not stunted, as shown in Table 1.

**Table 1:** Description of the study variables.

Variables	Mean ± SD	Min, Max	Frequency (Percentage)
Stunting			
Severely stunted			52 (19.48)
Moderately stunted			51 (19.10)
Normal (not stunted)			164 (61.42)
Mother’s year of schooling	5.79 ± 3.48	0,14	
Mothers’ education			
No education			46 (17.23)
Primary			88 (32.96)
Secondary			112 (41.95)
Higher secondary and more			21 (7.87)
Household size	3.78 ± 0.92	2, 8	
Sanitation facilities			
Unimproved			82 (30.71)
Improved			185 (69.29)
Birth Weight			
Less than or equal to 2.5 kg			71 (26.59)
Greater than 2.5 kg			196 (73.41)
ANC service			
Haven’t received			28 (10.49)
Received			239 (89.51)
Control over resources			
No			116 (43.45)
Yes			151 (56.55)
Caregiver knowledge about dietary diversity			
No			64 (23.97)
Yes			203 (76.03)
Mother’s nutrition knowledge (score)	0.57 ± 0.16	0.167, 1	
Mother’s nutrition knowledge			
Poor			117 (43.82)
Fair			131 (49.06)
Good			19 (7.12)
Child stay at daycare			
No			169 (63.3)
Yes			98 (36.7)

The mother’s year of schooling represents the number of years she attended school, and the average year of schooling for the mothers in the study was 5.79 years. This variable was further classified into different groups, with 17% of mothers having no education, 33% having up to primary education, 42% having up to secondary education, and the remaining 8% having higher secondary or higher levels of education.

Mothers’ nutritional knowledge mentioned above was assessed from the six nutrition-related questions which are represented below.

The study also found that the average household size was 3.78, with a minimum of 2 members. Additionally, 69% of families had improved sanitation facilities, and almost 73% of children were born with a birth weight of more than 2.5 kg.

In terms of healthcare, RMG workers rarely visited doctors and only sought medical attention in case of extreme emergencies. Nearly 90% of respondents visited doctors or health clinics two or fewer times to receive ANC services. Despite earning their own income, only 57% of respondents had control over their resources. When mothers went to work, they left their children with either relatives or non-relative caregivers. Almost 76% of these caregivers were aware of the child’s dietary diversity.

The mean value of mothers’ nutritional knowledge score was 0.57, which was further classified into three categories. Of the mothers, 44% had poor nutritional knowledge, 49% had fair knowledge, and the remaining 7% had good nutritional knowledge. Finally, daycare facilities in RMG industries in Bangladesh were found to be a recent addition. 37% of respondents’ children were placed in poorly managed daycare centres during their parents’ work hours.

Most of the mothers (92%) believed that only fat babies were not healthy (Table 1). On the other hand, the majority (97%) of the mothers knew that feeding colostrum to a baby was important, and nearly 82% of mothers introduced semi-solid foods to their child when their child turned 6 months old. Around 87% of mothers provided oral rehydration solution (ORS) as a fluid when their children were suffering from diarrhoea. About 58% of the mothers knew that they should consume fruits, vegetables, and other nutrition-rich foods every day. Around 87% of mothers had a negative perception of fast food and did not tend to provide it regularly to their children (Table 2). After summing up all the positive responses, the final score was divided by 6 so that the extent of mothers’ nutritional knowledge scale lies between the range of 0 to 1. Furthermore, it was multiplied by 100 for better understanding. With exception of ANC services, all the other variables were found significantly associated with childhood stunting at a different significance level through the Pearson chi-squared test. (Table 3).

**Table 2:** The extent of mothers’ nutritional knowledge.

Responses	No	Yes
Think fat baby is not healthy	20 (7.49)	247 (92.51)
Feeding colostrum to a baby is important	8 (3.00)	259 (97.00)
Introduce semi-solid foods to child	48 (17.98)	219 (82.02)
ORS should be provided who suffer from diarrhoea	34 (12.73)	233 (87.27)
At least 5 colours of food should we consume every day	113 (42.32)	154 (57.68)
Children should not regularly consume fast foods	34 (12.73)	233 (87.27)

Frequency (percentage).

**Table 3:** Distribution of stunting of ready-made garment workers’ children under the age of five according to different factors.

Variables	Stunting			Chi-square	P-value
	Severely stunted	Moderately stunted	Normal		
Mothers’ education					
No education	11 (23.91) (21.15)	14 (30.43) (27.45)	21 (45.65) (12.8)	14.77	0.0221
Primary	19 (21.59) (36.54)	10 (11.36) (19.61)	59 (67.05) (35.98)		
Secondary	19 (16.96) (36.54)	19 (16.96) (37.25)	74 (66.07) (45.12)		
Higher secondary and more	3 (14.29) (5.77)	8 (38.1) (15.69)	10 (47.62) (6.1)		
Sanitation facilities					
Unimproved	18 (21.95) (34.62)	22 (26.83) (43.14)	42 (51.22) (25.61)	6.08	0.0479
Improved	34 (18.38) (65.38)	29 (15.68) (56.86)	122 (65.95) (74.39)		
Birth Weight					
Less than or equal to 2.5 kg	19 (26.76) (36.54)	21 (29.58) (41.18)	31 (43.66) (18.9)	13.16	0.0014
Greater than 2.5 kg	33 (16.84) (63.46)	30 (15.31) (58.82)	133 (67.86) (81.1)		
ANC service					
Haven’t received	8 (28.57) (15.38)	8 (28.57) (15.69)	12 (42.86) (7.32)	4.55	0.1026
Received	44 (18.41) (84.62)	43 (17.99) (84.31)	152 (63.6) (92.68)		
Control over resources					
No	31 (26.72) (59.62)	19 (16.38) (37.25)	66 (56.9) (40.24)	7.01	0.03
Yes	21 (13.91) (40.38)	32 (21.19) (62.75)	98 (64.9) (59.76)		
Caregiver **knowledge** about dietary diversity					
No	20 (31.25) (38.46)	10 (15.63) (19.61)	34 (53.13) (20.73)	7.47	0.0239
Yes	32 (15.76) (61.54)	41 (20.2) (80.39)	130 (64.04) (79.27)		
Child stay at daycare					
No	32 (18.93) (61.54)	33 (19.53) (64.71)	104 (61.54) (63.41)	0.11	0.09447
Yes	20 (20.41) (38.46)	18 (18.37) (35.29)	60 (61.22) (36.59)		
Mother’s nutrition knowledge					
Poor	24 (20.51) (46.15)	24 (20.51) (47.06)	69 (58.97) (42.07)	1.33	0.08558
Fair	24 (18.32) (46.15)	25 (19.08) (49.02)	82 (62.6) (50)		
Good	4 (21.05) (7.69)	2 (10.53) (3.92)	13 (68.42) (7.93)		

Frequency (row relative percentage) (column relative percentage).

Row relative percentages were used to chi-square test and column relative percentages were used to describe the distribution of variables.

### Regression results

The study used relative risk ratio (RRR) to explain the result of the best-fitted regression model. Akaike information criteria (AIC) have been found lowest in model 4 and it has been chosen as the best-fitted model (Table 4). From the model, it was found that if mothers’ education shifts from no education to up to the primary, the child would be 0.211 (95% CI: 0.071–0.627) times more likely to have a normal weight and height than moderately stunted and if mothers’ education shifts from no education to up to secondary, then the child would be 0.384 (95% CI: 0.138–1.065) times more likely to have a normal weight and height than moderately stunted. When the household size increases by one person, the child would become 0.62 (95% CI: 0.401–0.965) times more likely to be normal than being severely stunted and 0.65 (95% CI: 0.423–1.02) times more likely to be normal than moderately stunted. When the family has an improved sanitation facility, the child would be 0.46 (95% CI: 0.208–1.06) times more likely to have normal weight and height than severely stunted and 0.31 (95% CI: 0.137–0.707) times more likely to have normal weight and height than moderately stunted (Table 3). Children with more than 2.5 kg birth weight would have 0.34 (95% CI: 0.159–0.751) times more likelihood to be normal than severely stunted and 0.31 (95% CI: 0.146–0.677) times more likely to be normal than moderately stunted. Mothers’ who received ANC during their pregnancy, their children would have 0.328 (95% CI: 0.105–1.026) times more likely of being normal than severely stunted and 0.23 (95% CI: 0.077–0.726) times more likely of being normal than moderately stunted. When mothers have control over their income, their child would become 0.26 (95% CI: 0.079–0.88) times more likely to be of normal weight and height than severely stunted. An increase in one unit of mothers’ nutritional knowledge score increases the chance for the child to be normal than moderately stunted by 0.059 (95% CI: 0.005–0.683) times (Table 4).

**Table 4:** Factors influencing stunting of ready-made garment workers’ children under the age of five: Multinomial logit regression model.

Stunting (normal)	RRR Sig (95% Confidence Interval)							
	Model 1		Model 2		Model 3		Model 4	
	AIC = 480.157		AIC = 483.25		AIC = 478.62		AIC = 474.882	
	Severely stunted	Moderately stunted	Severely stunted	Moderately stunted	Severely stunted	Moderately stunted	Severely stunted	Moderately stunted
Mother’s year of schooling			0.944	0.951	0.954	0.974		
			(0.845–1.054)	(0.857–1.056)	(0.854–1.067)	(0.875–1.083)		
Mother’s education (no education)								
Up to primary	0.518	0.2***					0.522	0.211***
	(0.18–1.491)	(0.067–0.594)					(0.181–1.505)	(0.071–0.627)
Up to secondary	0.5	0.316**					0.539	0.384*
	(0.169–1.483)	(0.116–0.863)					(0.18–1.613)	(0.138–1.065)
Higher secondary	0.445	0.77					0.503	0.999
	(0.078–2.527)	(0.187–3.18)					(0.088–2.876)	(0.237–4.205)
Household size	0.62**	0.643**	0.611**	0.63**	0.616**	0.64**	0.622**	0.657*
	(0.4–0.962)	(0.414–0.999)	(0.394–0.948)	(0.41–0.968)	(0.397–0.954)	(0.417–0.983)	(0.401–0.965)	(0.423–1.02)
Improved sanitation practice (unimproved)								
Improved	0.469*	0.29***	0.468*	0.308***	0.477*	0.33***	0.469*	0.311***
	(0.206–1.067)	(0.128–0.672)	(0.209–1.047)	(0.141–0.674)	(0.216–1.057)	(0.153–0.715)	(0.208–1.06)	(0.137–0.707)
Birth weight (less than or equal 2.5 kg)								
Greater than 2.5 kg	0.34***	0.29***	0.324***	0.267***	0.33***	0.291***	0.345***	0.314***
	(0.155–0.746)	(0.132–0.625)	(0.148–0.707)	(0.125–0.568)	(0.152–0.715)	(0.138–0.61)	(0.159–0.751)	(0.146–0.677)
Antenatal care (did not receive)								
Received	0.334*	0.241**	0.341*	0.286**	0.336*	0.28**	0.328*	0.237**
	(0.107–1.043)	(0.078–0.74)	(0.11–1.053)	(0.096–0.851)	(0.108–1.039)	(0.095–0.83)	(0.105–1.026)	(0.077–0.726)
Control over resources (no)								
Yes	0.228**	1.519	0.221***	1.607	0.254**	1.887	0.264**	1.942
	(0.072–0.723)	(0.362–6.379)	(0.071–0.688)	(0.367–7.038)	(0.078–0.827)	(0.464–7.679)	(0.079–0.88)	(0.47–8.031)
Caregiver’s knowledge about dietary diversity (no)								
Yes	0.442**	0.843	0.451**	0.89	0.42**	0.854	0.407**	0.818
	(0.2–0.976)	(0.34–2.091)	(0.206–0.988)	(0.37–2.14)	(0.191–0.926)	(0.356–2.049)	(0.183–0.906)	(0.329–2.034)
Mother’s nutrition knowledge					0.302	0.102*	0.275	0.059**
					(0.028–3.315)	(0.01–1.043)	(0.024–3.124)	(0.005–0.683)
Mother’s nutrition knowledge (category) (poor)								
Fair	0.992	0.92	1.027	1.064				
	(0.448–2.196)	(0.43–1.969)	(0.464–2.276)	(0.509–2.221)				
Good	0.577	0.189*	0.577	0.262				
	(0.141–2.362)	(0.03–1.178)	(0.142–2.348)	(0.046–1.488)				
Child stay at daycare (no)								
Yes	0.284*	1.257	0.263**	1.14	0.317*	1.47	0.338	1.724
	(0.078–1.033)	(0.284–5.558)	(0.076–0.91)	(0.254–5.119)	(0.091–1.106)	(0.355–6.092)	(0.092–1.248)	(0.398–7.477)
Constant	206.3***	74.2***	178.2***	32.138**	282.746***	72.531***	362.95***	192.09***
	(14.868–2861.70)	(4.471–1233.1)	(13.663–2324.83)	(2.211–467.039)	(17.591–4544.64)	(4.156–1265.8)	(20.442–6444.169)	(9.321–3958.511)

***1%, **5%, *10% level of significance; Source: Authors’ calculation.

## Discussion

In general, and since time immemorial, mothers have been the principal household caregivers, especially for infants and children, throughout the world. Such caregiving responsibility became more and more challenging for contemporary working mothers, given their multiple burdens of responsibilities. In this case, the RMG women workers in Bangladesh have to work for long hours in the factories which in turn severely affects the health and nutrition outcomes for themselves as well as for their children. In line with this, many of the RMG women workers had the experience of early marriages, they lacked access to ANC services and hardly visited doctors, hospitals and healthcare centres [73]. The study found that as a mother’s educational attainment increases, her child is less likely to be stunted. Evidence suggests that both mothers’ formal education and nutritional knowledge play an important role in increasing the physical growth and cognitive development of under-five children. Most of the RMG workers received lower levels of education, for instance, about 40% of our respondents had a secondary or higher secondary level of education. Moreover, the questionable quality of the education they receive raises additional concerns in this regard. Literature also suggests that educated mothers have a better quality of life, are more likely to have insights on child diet, receive ANC, and achieve greater physical and financial freedom [8,18,20,21,27–29,35,37,73–75]. Educated women often have autonomy with regard to their personal life, household decisions, resource use, and dignity in their communities [73]. Some authors noted that a mother’s formal education is an insignificant factor in a child nutritional status [21,30–32], while Hamel et al. [34] indicated that a mother’s practical knowledge about child nutrition might be more important than formal maternal education for child nutritional outcomes. This study also found that as the nutritional knowledge of a mother increases, a child would be less likely to be moderately stunted. Similarly, a caregiver’s knowledge of child nutrition plays a significant role in reducing severe stunting. Additionally, if mothers have control over household resources, the child would be more likely to be healthy. Economic freedom may also enable them to decide on buying nutritious foods, availing better housing with improved sanitation and seeking health services. Their control over income may also allow them to buy mechanical gadgets to reduce the time burden of household chores and to share some of the time with their children. However, we found that within the reproductive time, RMG women workers spend relatively more time cooking as they have to perform almost all the work manually. Smith et al. [76] also found that control over financial resources was associated with improvements in child nutrition, while McKenna et al. [67] did not find any significant relationship between financial decision-making power and stunting or wasting in children.

This study shows that ANC plays a significant role in improving a child’s nutritional status, similar to the findings of some previous studies [26,34,35]. If a mother received proper ANC during her pregnancy, the child was less likely to suffer from stunting. Two types of care are typically provided under ANC services: clinical care and counselling that provides nutritional advisory guidelines to pregnant women. Undoubtedly, both play a significant role in ensuring long-term health and nutrition outcomes for the child. Increased access to ANC warrants better physical growth of a child during pregnancy and healthier birth weight, and this may aid in the future weight gain and physical growth of a child. There is a strong intergenerational relationship between birth weight and subsequent gain of length as rationalized by both genetic and environmental influences across generations [25,77]. It could be mentioned here that the RMG workers have minimal access to the ANC services due to their time limitations from high work pressure.

Our study found that children who were born with lower birth weights (LBW) were more likely to be stunted [40]; who also mentioned that LBW was the significant and prominent determinant of stunting after adjusting for other factors such as age and sex, and; children with a history of LBW had 3-fold greater odds of being stunted compared to children with normal birth weight. They also found that the prevalence of LBW was about 29% and approximately half of the children were stunted by the age of 24 months. A study found LBW as a substantial negative predictor for childhood mortality and morbidity [78]. Another study mentioned that in developing countries, LBW is often associated with intrauterine growth restriction (IUGR) and stunting [79]. Likewise, despite a marked improvement in other health-related indicators, Bangladesh is struggling to tackle the burden of chronic malnutrition. Long ago it has reported that birth weight as the key determinant of subsequent growth status during infancy based on a study conducted in a Bangladeshi slum area [80]. Similar to [58], our study also found that once a baby is born underweight, the risk of becoming undernourished increases.

Proper sanitation plays an important role in improving a child as well as household health status. Improper sanitation may lead to the prevalence of soil-transmitted helminths which affect the gastrointestinal tract of a child induced by fecal contamination that can result in impairment in physical development [81]. Most of the RMG workers who accompany their children stay in a crowded dwelling with a shared toilet facility available for 5 to 10 families or even more. Moreover, RMG workers do not have much time to look after their children, which impedes proper and safe sanitation practices. Our results found that children from households with proper sanitation were less likely to be stunted. This may be because proper sanitation can reduce the risk of diarrhoea which reduces the risk of long-term malnutrition [3,20,48–53].

When the parents are away from home for work purposes, the relatives or non-relative caregivers take care of the child. In developed countries, child daycare facilities exist at an affordable cost regardless of socioeconomic class. However, in Bangladesh, childcare facilities are hardly available. Recently, RMG industries have started to introduce it but the quality is very low. As a result, most of the RMG workers do not accompany their children with them as they cannot afford to attend to any relative to take care of the child. As a result, many workers leave their children at their origin. However, if any adult family relative stays with the child when the parents are away, it may minimize the negative outcome of the child’s nutritional status. We found that children from large families are less likely to be stunted as there are typically other people to take care of the child. This is in line with the previous study [59,60]. However, there may also be risks to food insecurity within the family when there are more members, which could reverse child nutrition [32,54–58] that can be abated by increasing household income. Yet, it is challenging to maintain a large family with a small income of RMG workers. Thus, an affordable and proper daycare facility could help to ensure adequate nutrition and well-being for the children of RMG women workers in Bangladesh.

### Policy implications

The study found that many RMG workers are malnourished and lack proper ANC during pregnancy. This highlights the need for ANC services to improve mothers’ health and knowledge, which can lead to improved health outcomes for both mother and child. However, RMG workers often lack the time and awareness to access healthcare services, and pregnant workers may be afraid of losing their jobs if they disclose their pregnancy. To address these issues, the government and BGMEA should work together to introduce ANC facilities and mother-friendly organizational rules and legal protections. Counselling for husbands and family members can also increase household cohesiveness.

Improving the socioeconomic status of RMG workers can also lead to better health outcomes for their children. Many RMG households cannot afford proper food and healthcare for their children, leading to stunting and other negative health outcomes. Safety net programs like cash and food transfers can decrease household poverty-related stress and improve their living conditions. Additionally, RMG workers often cannot find suitable childcare arrangements, so the industry should establish quality childcare facilities with trained staff to support working mothers. As the RMG sector continues to expand, it is crucial to provide support and services to protect the health and well-being of these workers and their families.

## Conclusions

The "Made in Bangladesh" tag has brought Bangladesh to the attention of the world. However, this industry relies on millions of resource-constrained and predominantly migrant females, who often have to leave their children to work long hours, making it difficult for them to care for their children. The unanswered strategic questions are: what is the nutritional status of RMG workers’ children? How are they growing up? What role does the state play in the future of these citizens? The study’s findings suggest that we need to improve the socioeconomic status of mothers working in the RMG industry to improve the nutritional status of their children, who belong to an underprivileged segment of society. To achieve this, rigorous policy interventions are required. Our study results suggest that context-specific strategies should be formulated to reduce growth uncertainty and negative health outcomes.

## Supporting information

S1 File (XLSX)

S2 File (DOCX)

## Abbreviations

AIC: Akaike information criterion
ANC: Antenatal care
BMI: Body mass index
CDC: Centers for Disease Control and Prevention
CI: Confidence interval
CPD: Center for Policy Dialogue
FANTA: Food and Nutrition Technical Assistance III Project
FAO: Food and Agricultural Organization
HAZ: Height for age Z score
ORS: Oral saline
RMG: Ready-made garments
UNICEF: United Nations Children’s Fund
USAID: United State Agency for International Development
WHO: World Health Organization
