# Supplementary material for: Nutritional status of under-five aged children of ready-made garment workers in Bangladesh: A cross-sectional study
Source: PLoS One. 2023 Apr 13;18(4):e0284325. doi: 10.1371/journal.pone.0284325 (PMC10101446; doi:10.1371/journal.pone.0284325)
Supplement: S2 File — (DOCX) [file pone.0284325.s002.docx]

**Model 1**

Hausman tests of IIA assumption (N=267)
 Ho: Odds(Outcome-J vs Outcome-K) are independent of other alternatives

|  | chi2 | df | P>chi2 |
| --- | --- | --- | --- |

| Severely | -4.201 | 13 | . |
| --- | --- | --- | --- |

| Moderately | -4.077 | 13 | . |
| --- | --- | --- | --- |

| Normal | 4.134 | 13 | 0.990 |
| --- | --- | --- | --- |

Note: A significant test is evidence against Ho.
 Note: If chi2<0, the estimated model does not meet asymptotic assumptions.

suest-based Hausman tests of IIA assumption (N=267)
 Ho: Odds(Outcome-J vs Outcome-K) are independent of other alternatives

|  | chi2 | df | P>chi2 |
| --- | --- | --- | --- |

| Severely | 9.330 | 13 | 0.748 |
| --- | --- | --- | --- |

| Moderately | 7.831 | 13 | 0.854 |
| --- | --- | --- | --- |

| Normal | 9.256 | 13 | 0.753 |
| --- | --- | --- | --- |

Note: A significant test is evidence against Ho.

Small-Hsiao tests of IIA assumption (N=267)
 Ho: Odds(Outcome-J vs Outcome-K) are independent of other alternatives

|  | df |
| --- | --- |

| Severely | -65.549 | -58.795 | 13.507 | 13 | 0.409 |
| --- | --- | --- | --- | --- | --- |

| Moderately | -80.380 | -59.961 | 40.837 | 13 | 0.000 |
| --- | --- | --- | --- | --- | --- |

| Normal | -41.742 | -33.988 | 15.509 | 13 | 0.277 |
| --- | --- | --- | --- | --- | --- |

Note: A significant test is evidence against Ho.

**Model 2**

Hausman tests of IIA assumption (N=267)
 Ho: Odds(Outcome-J vs Outcome-K) are independent of other alternatives

|  | chi2 | df | P>chi2 |
| --- | --- | --- | --- |

| Severely | -1.711 | 11 | . |
| --- | --- | --- | --- |

| Moderately | 0.403 | 11 | 1.000 |
| --- | --- | --- | --- |

| Normal | 1.311 | 11 | 1.000 |
| --- | --- | --- | --- |

Note: A significant test is evidence against Ho.
 Note: If chi2<0, the estimated model does not meet asymptotic assumptions.

suest-based Hausman tests of IIA assumption (N=267)
 Ho: Odds(Outcome-J vs Outcome-K) are independent of other alternatives

|  | chi2 | df | P>chi2 |
| --- | --- | --- | --- |

| Severely | 6.965 | 11 | 0.802 |
| --- | --- | --- | --- |

| Moderately | 7.121 | 11 | 0.789 |
| --- | --- | --- | --- |

| Normal | 6.099 | 11 | 0.867 |
| --- | --- | --- | --- |

Note: A significant test is evidence against Ho.

Small-Hsiao tests of IIA assumption (N=267)
 Ho: Odds(Outcome-J vs Outcome-K) are independent of other alternatives

|  | df |
| --- | --- |

| Severely | -50.579 | -46.474 | 8.209 | 11 | 0.695 |
| --- | --- | --- | --- | --- | --- |

| Moderately | -59.284 | -49.846 | 18.877 | 11 | 0.063 |
| --- | --- | --- | --- | --- | --- |

| Normal | -32.206 | -26.402 | 11.608 | 11 | 0.394 |
| --- | --- | --- | --- | --- | --- |

Note: A significant test is evidence against Ho.

**Model 3**

Hausman tests of IIA assumption (N=267)
 Ho: Odds(Outcome-J vs Outcome-K) are independent of other alternatives

|  | chi2 | df | P>chi2 |
| --- | --- | --- | --- |

| Severely | -5.282 | 10 | . |
| --- | --- | --- | --- |

| Moderately | -3.087 | 10 | . |
| --- | --- | --- | --- |

| Normal | 2.410 | 10 | 0.992 |
| --- | --- | --- | --- |

Note: A significant test is evidence against Ho.
 Note: If chi2<0, the estimated model does not meet asymptotic assumptions.

suest-based Hausman tests of IIA assumption (N=267)
 Ho: Odds(Outcome-J vs Outcome-K) are independent of other alternatives

|  | chi2 | df | P>chi2 |
| --- | --- | --- | --- |

| Severely | 10.107 | 10 | 0.431 |
| --- | --- | --- | --- |

| Moderately | 8.931 | 10 | 0.539 |
| --- | --- | --- | --- |

| Normal | 7.024 | 10 | 0.723 |
| --- | --- | --- | --- |

Note: A significant test is evidence against Ho.

Small-Hsiao tests of IIA assumption (N=267)
 Ho: Odds(Outcome-J vs Outcome-K) are independent of other alternatives

|  | df |
| --- | --- |

| Severely | -56.470 | -54.384 | 4.172 | 10 | 0.939 |
| --- | --- | --- | --- | --- | --- |

| Moderately | -46.840 | -42.343 | 8.995 | 10 | 0.533 |
| --- | --- | --- | --- | --- | --- |

| Normal | -30.405 | -26.918 | 6.972 | 10 | 0.728 |
| --- | --- | --- | --- | --- | --- |

Note: A significant test is evidence against Ho.

**Model 4**

Hausman tests of IIA assumption (N=267)
 Ho: Odds(Outcome-J vs Outcome-K) are independent of other alternatives

|  | chi2 | df | P>chi2 |
| --- | --- | --- | --- |

| Severely | -5.373 | 12 | . |
| --- | --- | --- | --- |

| Moderately | -2.198 | 12 | . |
| --- | --- | --- | --- |

| Normal | 4.621 | 12 | 0.969 |
| --- | --- | --- | --- |

Note: A significant test is evidence against Ho.
 Note: If chi2<0, the estimated model does not meet asymptotic assumptions.

suest-based Hausman tests of IIA assumption (N=267)
 Ho: Odds(Outcome-J vs Outcome-K) are independent of other alternatives

|  | chi2 | df | P>chi2 |
| --- | --- | --- | --- |

| Severely | 10.709 | 12 | 0.554 |
| --- | --- | --- | --- |

| Moderately | 9.730 | 12 | 0.640 |
| --- | --- | --- | --- |

| Normal | 9.673 | 12 | 0.645 |
| --- | --- | --- | --- |

Note: A significant test is evidence against Ho.
Small-Hsiao tests of IIA assumption (N=267)
 Ho: Odds(Outcome-J vs Outcome-K) are independent of other alternatives

|  | df |
| --- | --- |

| Severely | -49.928 | -43.371 | 13.114 | 12 | 0.361 |
| --- | --- | --- | --- | --- | --- |

| Moderately | -57.399 | -50.433 | 13.933 | 12 | 0.305 |
| --- | --- | --- | --- | --- | --- |

| Normal | -36.800 | -26.750 | 20.100 | 12 | 0.065 |
| --- | --- | --- | --- | --- | --- |

Note: A significant test is evidence against Ho.
